# Are retinol, vitamin C, vitamin E, folate and carotenoids intake associated with bladder cancer risk? Results from the Netherlands Cohort Study

**DOI:** 10.1054/bjoc.2001.1968

**Published:** 2001-09

**Authors:** M P A Zeegers, R A Goldbohm, P A van den Brandt

**Affiliations:** Department of Epidemiology, Maastricht University, Maastricht; Department of Nutritional Epidemiology, TNO Nutrition and Food Research, Zeist, The Netherlands

**Keywords:** urologic neoplasms, bladder neoplasms, vitamin-A, vitamin-C, vitamin-E, folate, carotenoids

## Abstract

In the Netherlands Cohort Study among 120 852 subjects aged 55–69 years at baseline (1986), the association between vitamins and carotenoids intake, vitamin supplement use, and bladder cancer incidence was examined. Exposure status was measured with a food-frequency questionnaire. After 6.3 years of follow-up, data from 569 cases and 3123 subcohort members were available for case-cohort analyses. The age-, sex-, and smoking-adjusted relative risks (RRs) for retinol, vitamin E, folate, a-carotene, b-carotene, lutein and zeaxanthin, and lycopene were 1.04, 0.98, 1.03, 0.99, 1.16, 1.11, and 1.08, respectively, comparing highest to lowest quintile of intake. Only vitamin C (RR: 0.81, 95% CI: 0.61–1.07, P-trend = 0.08), and b-cryptoxanthin intake (RR: 0.74, 95% CI: 0.53–1.03, P-trend < 0.01) were inversely associated with bladder cancer risk. The association with vitamin C disappeared after adjustment for b-cryptoxanthin but not vice versa. The RRs for supplemental use of vitamin A, C or E compared to no use were around unity.   We conclude that dietary or supplemental intake of vitamin A, vitamin C, vitamin E, and intake of folate, and most carotenoids are not associated with bladder cancer. In this study, only b-cryptoxanthin intake appeared to be inversely associated. © 2001 Cancer Research Campaignhttp://www.bjcancer.com


*British Journal of Cancer* (2001) **85**(7), 977–983
© 2001 Cancer Research Campaign
doi: 10.1054/ bjoc.2001.1968, available online at http://www.idealibrary.com on IDEAL

### Are retinol, vitamin C, vitamin E, folate and carotenoids intake associated with bladder cancer risk? Results from the Netherlands Cohort Study

**MPA Zeegers[1], RA Goldbohm[2] and PA van den Brandt[1]**

[1]Department of Epidemiology, Maastricht University, Maastricht; and [2]Department of Nutritional Epidemiology, TNO Nutrition, and Food Research, Zeist, The Netherlands

**Summary** In the Netherlands Cohort Study among 120 852 subjects aged 55–69 years at baseline (1986), the association between vitamins and carotenoids intake, vitamin supplement use, and bladder cancer incidence was examined. Exposure status was measured with a food-frequency questionnaire. After 6.3 years of follow-up, data from 569 cases and 3123 subcohort members were available for case-cohort analyses. The age-, sex-, and smoking-adjusted relative risks (RRs) for retinol, vitamin E, folate, α-carotene, β-carotene, lutein and zeaxanthin, and lycopene were 1.04, 0.98, 1.03, 0.99, 1.16, 1.11, and 1.08, respectively, comparing highest to lowest quintile of intake. Only vitamin C (RR: 0.81, 95% CI: 0.61–1.07, *P*-trend = 0.08), and β-cryptoxanthin intake (RR: 0.74, 95% CI: 0.53–1.03, *P*-trend < 0.01) were inversely associated with bladder cancer risk. The association with vitamin C disappeared after adjustment for β-cryptoxanthin but not vice versa. The RRs for supplemental use of vitamin A, C or E compared to no use were around unity.

We conclude that dietary or supplemental intake of vitamin A, vitamin C, vitamin E, and intake of folate, and most carotenoids are not associated with bladder cancer. In this study, only β-cryptoxanthin intake appeared to be inversely associated. © 2001 Cancer Research Campaign http://www.bjcancer.com

**Keywords**: urologic neoplasms; bladder neoplasms; vitamin-A; vitamin-C; vitamin-E; folate; carotenoids

Bladder cancer is the most common urological cancer, and is the seventh most common cancer among men, accounting for approximately 200 000 new cases per year worldwide (Parkin et al, 1999). Over the last four decades, many epidemiologic studies and several reviews have been conducted to investigate determinants of bladder cancer (Johansson and Cohen, 1997; Ross et al, 1996; Silverman et al, 1992; van der Meijden, 1998; World Cancer Research Fund & American Institute for Cancer Research, 1997). These studies suggested that bladder cancer is influenced by environmental factors, including cigarette smoking, fluid consumption, schistosomal infections, exposure to industrial chemicals (e.g. aromatic amines) and diet.

Among the dietary factors, it has been suggested that vegetable and fruit consumption may protect against cancer at various anatomical sites. With respect to bladder cancer, most epidemiological studies support a possible protective effect of vegetable (Bruemmer et al, 1996; D'Avanzo et al, 1995; Michaud et al, 1999; Mills et al, 1991; Negri et al, 1991) and fruit consumption (Bruemmer et al, 1996; Chyou et al, 1993; Negri et al, 1991; Shibata et al, 1992), although some studies have reported no association for vegetable (Riboli et al, 1991; Shibata et al, 1992) or fruit consumption (Michaud et al, 1999; Riboli et al, 1991). There are many candidate agents in fruits and vegetables that influence bladder cancer risk, including carotenoids, vitamin C, vitamin E, and folate.

*Received 30 January 2001*
*Revised 5 June 2001*
*Accepted 5 June 2001*

*Correspondence to:* MPA Zeegers

In the present prospective study, we were able to explore the influence of the intake of different vitamins (i.e. retinol, vitamin C, vitamin E, and folate), and the use of vitamin-containing supplements on bladder cancer risk with substantially more cases than in previous studies. Furthermore, with the use of a recently developed food composition table on carotenoids in Dutch foods (1986), analyses were performed with α-carotene, β-carotene, lutein plus zeaxanthin, β-cryptoxanthin, and lycopene.

### MATERIALS AND METHODS

### Cohort

The study design has been described in detail previously (van den Brandt et al, 1990a). The cohort includes 58 279 men and 62 573 women aged 55–69 years at baseline (1986). The study population originated from 204 municipal population registries throughout the country. The case cohort approach was used for data processing and analysis (Prentice, 1986). Following this approach, a subcohort of 3500 subjects (1688 men, 1812 women) was randomly sampled from the cohort after baseline exposure measurement. The subcohort has been followed up for vital status information. No subcohort members were lost to follow-up.

### Follow-up

Follow-up for incident cancer was established by record linkage to cancer registries and the Dutch national database of pathology reports (van den Brandt et al, 1990b). The completeness of cancer follow-up was estimated to be over 95% (Goldbohm et al, 1994b). The presented analysis is restricted to cancer incidence in 6.3 years of follow-up. After excluding prevalent cases with cancer other than skin cancer a total of 3346 subcohort members (1630 men and 1716 women) and 619 incident cases (532 men and 87 women) with microscopically confirmed, incident carcinomas of the urinary bladder, ureters, renal pelvis or urethra were identified. Of these cases, 584 (94.3%) were diagnosed with bladder cancer of which 559 (95.7%) were transitional cell carcinomas. Because the overwhelming majority of tumours occurred in the urinary bladder and the ureters, renal pelvis, and urethra are covered by the same urothelium as the urinary bladder, the term bladder cancer is used as a synonym for these neoplasms.

### Exposure status

At baseline, the cohort members completed a mailed, self-administered questionnaire on risk factors for cancer. Usual consumption of food and beverages during the year preceding the start of the study was assessed with a 150-item semiquantitative food frequency questionnaire. The questionnaire was validated against a 9-day diet record. The energy and sex-adjusted Pearson correlation coefficients between the 9-day record and the questionnaire were 0.48 for total vitamin A and 0.55 for vitamin C (Goldbohm et al, 1994a). The questionnaire data were key entered twice and processed in a standardized manner blinded with respect to case/subcohort status in order to minimize observer bias in coding and interpretation of the data. In this study, variables of principle interest were retinol, vitamins C and E, folate, several carotenoids and the use of vitamin-containing supplements. The mean daily intake of retinol, vitamins C and E were calculated using the computerized Dutch Food Composition Table (1986). Folate data were derived from a validated liquid chromatography trienzyme method with which the most important Dutch foods contributing to folate intake were analysed (Konings, 1999). For calculating the intake of specific carotenoids an additional food composition table has been constructed recently (Goldbohm et al, 1998). Briefly, regularly eaten vegetables were comprehensively sampled and analysed for their carotenoid content and the database was completed with data from the recent literature and information from food manufacturers (Goldbohm et al, 1998). With this carotenoid table, we were able to evaluate six of the most important types of carotenoids: α-carotene, β-carotene, lutein, zeaxanthin, β-cryptoxanthin and lycopene. In the carotenoid food composition table, lutein and zeaxanthin were combined, because most literature sources had not distinguished these two carotenoids. Most vegetables, however, contain primarily lutein and only minor amounts of zeaxanthin. Information on dietary supplement use was collected using an open-ended question with space for adding a maximum of four different supplements. Participants were asked whether they used vitamin tablets, drops or other preparations during five years before baseline. Subjects were categorized in users or non-users of supplements containing vitamin A, C or E.

### Statistical analyses

Subjects with incomplete or inconsistent dietary data were excluded leaving 569 cases (491 men and 78 women) with bladder cancer and 3123 subcohort members (1525 men and 1598 women) for the analyses.

Incidence rate ratios (RR) and corresponding 95% confidence intervals (CI) for bladder cancer were estimated using exponentially distributed failure time regression models (Volovics and van den Brandt, 1997) with the Stata statistical software package (StataCorp, 1999) in which was accounted for additional variance introduced by sampling from the cohort (Barlow, 1994; Lin and Ying, 1993).

Subjects were classified by quintile of intake of the vitamins and carotenoids or were classified by their use of vitamin-containing supplements (yes/no). For vitamin C, quintiles 2 and 3 and quintiles 4 and 5 were combined, because the validation study demonstrated that these quintiles could not be distinguished (Goldbohm et al, 1994a). Tests for trends in risk for bladder cancer over multiple categories were assessed by fitting ordinal exposure variables as continuous terms and performing likelihood-ratio tests between regression models with and without these variables.

The following variables were subsequently considered as potential confounders based on earlier analyses (Steinmaus et al, 2000; Zeegers et al, 1999; 2000a; 2000b): consumption of alcohol (g/day), coffee (ml/day), tea (ml/day) water (ml/day), vegetables (g/day) and fruits (g/day), current cigarette smoking (yes/no), smoking amount (cigarettes/day), smoking duration (years of cigarette smoking), occupational exposure to dye, rubber, leather or vehicle fumes (ever/never), and first degree family history of bladder cancer (yes/no). Those variables that showed a more than 10% influence on the risk of bladder cancer in a multivariable model were included as covariates in the analyses. For β-crypthoxanthin intake, subgroup analyses were performed after stratification by tumour morphology and invasiveness using list wise deletion of missing data.

### RESULTS

Table 1 presents the distribution of the dietary and supplemental vitamin intakes and the distribution of potential confounding factors among bladder cancer cases and sub-cohort members. The dietary intakes of retinol, vitamin E, folate, α-carotene, β-carotene, lycopene, β-cryptoxanthin, and lutein/zeaxantine were comparable between cases and sub-cohort members. The intake of vitamin C and the use of supplements containing vitamin A, C or E were higher among sub-cohort members than among cases. Of the potential confounders, the distribution of age, total water, alcohol, coffee, tea, total vegetable, and total fruit consumption was similar for cases and sub-cohort members. More than 80% of the cases were male, whereas in the sub-cohort approximately 50% were male. Cigarette use was more often reported in cases than in sub-cohort members and smoking cases appeared to smoke more cigarettes per day for more years than smoking sub-cohort members. Less than 2% of the cases and sub-cohort members reported a family history of bladder cancer or had ever worked in a high-risk occupation (Table 1).

The association between vitamin and carotenoid intake and bladder cancer risk was similar in men and women for all vitamins and carotenoids (data not shown). Therefore, the results are presented for men and women combined. As is shown in Table 2, except for vitamin C intake, none of the vitamins (retinol, vitamin E, and folate), appeared to be associated with bladder cancer risk. The age-and sex-adjusted RRs of bladder cancer for quintiles 2 and 3 combined and quintiles 4 and 5 combined of vitamin C intake were 0.77 (CI: 0.61–0.98) and 0.74 (CI: 0.58–0.95) compared to the lowest quintile of intake, respectively (*P*-trend < 0.01). Although cigarette smoking amount and cigarette smoking duration are important determinants of bladder cancer, adjustment

 *© 2001 Cancer Research Campaign*

**Table 1** Description of mean daily intake of vitamins, carotenoids and vitamin supplement use and distribution of potential confounding factors, Netherlands Cohort Study (1986–1992)

| | Cases (*n* = 569) | | Sub-cohort (*n* = 3123) | |
|---|---|---|---|---|
| **Exposure variables** | **Mean** | **SD** | **Mean** | **SD** |
| Retinol (mg/day) | 0.59 | 0.33 | 0.54 | 0.31 |
| Vitamin C (mg/day) | 97.0 | 44.1 | 103 | 43.0 |
| Vitamin E (mg/day) | 13.9 | 6.27 | 13.4 | 6.24 |
| Folate (mg/day) | 219 | 73.7 | 212 | 0.70 |
| α-carotene (mg/day) | 0.66 | 0.48 | 0.70 | 0.58 |
| β-carotene (mg/day) | 2.89 | 1.42 | 2.97 | 1.58 |
| Lycopene (mg/day) | 1.14 | 1.94 | 1.19 | 1.74 |
| β-cryptoxanthin (mg/day) | 0.15 | 0.20 | 0.18 | 0.17 |
| Lutein/zeaxanthin (mg/day) | 2.54 | 1.23 | 2.52 | 1.11 |
| | Number | Percentage | Number | Percentage |
| Use of supplements containing vitamin A (% yes) | 30 | 5.27 | 226 | 7.24 |
| Use of supplements containing vitamin C (% yes) | 48 | 8.44 | 325 | 10.41 |
| Use of supplements containing vitamin E (% yes) | 28 | 4.92 | 204 | 6.53 |
| Potential confounding factors | Mean | SD | Mean | SD |
| Age (years) | 62.7 | 4.07 | 61.4 | 4.22 |
| Total water consumption (1/day) | 2.13 | 0.52 | 2.07 | 0.49 |
| Alcohol consumption (g/day) | 18.2 | 18.2 | 13.3 | 14.9 |
| Coffee consumption (cups/day) | 4.80 | 2.39 | 4.29 | 2.16 |
| Tea consumption (cups/day) | 2.45 | 2.02 | 2.86 | 2.03 |
| Total vegetable consumption (g/day) | 190 | 82.4 | 194 | 83.5 |
| Total fruit consumption (g/day) | 151 | 200 | 177 | 117 |
| Cigarette smoking amount (cig/day)* | 18.7 | 11.3 | 15.0 | 10.3 |
| Cigarette smoking duration (years)* | 37.5 | 11.6 | 31.6 | 12.3 |
| | Number | Percentage | Number | Percentage |
| Sex (% male) | 491 | 86.3 | 1525 | 48.8 |
| Cigarette smoking status (% current) | 255 | 44.8 | 875 | 28.0 |
| Cigarette smoking status (% never) | 62 | 10.9 | 1147 | 37.7 |
| Family history of bladder cancer (% yes) | 7 | 1.23 | 59 | 1.89 |
| High risk occupation (% yes)† | 4 | 0.70 | 15 | 0.48 |

*Among smokers; †ever exposed to dye, rubber, leather, and vehicle fumes.

**Table 2** Incidence rate ratios and 95% confidence intervals for bladder cancer according to quintiles of intake of retinol, vitamins C and E, and folate, Netherlands Cohort Study (1986–1992)

| | | Quintiles of intake | | | | | |
|---|---|---|---|---|---|---|---|
| **Vitamins** | **Cases** | **1 (low)*** | **2** | **3** | **4** | **5 (high)** | ***P*-trend** |
| Retinol | | | | | | | |
| Median intake (mg/day) | | 0.26 | 0.38 | 0.48 | 0.61 | 0.87 | |
| RR (95% CI)† | 569 | 1.00 | 0.75 (0.54–1.06) | 0.95 (0.69–1.30) | 0.98 (0.72–1.33) | 0.92 (0.68–1.26) | 0.71 |
| RR (95% CI)‡ | 475 | 1.00 | 0.83 (0.56–1.24) | 1.04 (0.72–1.51) | 1.02 (0.71–1.46) | 1.04 (0.72–1.48) | 0.67 |
| Vitamin C§ | | | | | | | |
| Median intake (mg/day) | | 54.9 | 86.6 | | 135 | | |
| RR (95% CI)† | 569 | 1.00 | 0.77 (0.61–0.98) | | 0.74 (0.58–0.95) | | < 0.01 |
| RR (95% CI)‡ | 475 | 1.00 | 0.87 (0.66–1.14) | | 0.81 (0.61–1.07) | | 0.08 |
| Vitamin E | | | | | | | |
| Median intake (mg/day) | | 6.59 | 9.27 | 12.2 | 15.8 | 21.7 | |
| RR (95% CI)† | 569 | 1.00 | 0.99 (0.72–1.36) | 1.08 (0.79–1.48) | 1.05 (0.77–1.43) | 0.88 (0.65–1.20) | 0.46 |
| RR (95% CI)‡ | 475 | 1.00 | 1.10 (0.76–1.61) | 1.28 (0.90–1.84) | 1.11 (0.77–1.59) | 0.98 (0.68–1.41) | 0.10 |
| Folate | | | | | | | |
| Median intake (mg/day) | | 0.14 | 0.17 | 0.20 | 0.23 | 0.29 | |
| RR (95% CI)† | 569 | 1.00 | 0.80 (0.58–1.10) | 0.80 (0.59–1.08) | 0.72 (0.53–0.98) | 0.93 (0.70–1.25) | 0.55 |
| RR (95% CI)‡ | 475 | 1.00 | 0.85 (0.59–1.23) | 0.87 (0.62–1.24) | 0.82 (0.58–1.17) | 1.03 (0.73–1.44) | 0.77 |

*Reference category; †adjusted for age (years) and sex; ‡adjusted for age (years); sex, cigarette smoking amount (cig/day) and cigarette smoking duration (years); §for vitamin C, quintiles 2 and 3 and quintiles 4 and 5 were combined.

*© 2001 Cancer Research Campaign*

**Table 3** Incidence rate ratios and 95% confidence intervals for bladder cancer according to quintiles of intake of carotenoids, Netherlands Cohort Study (1986–1992)

| Carotenoids | No. of cases | Quintiles of intake 1 (low)* | 2 | 3 | 4 | 5 (high) | *P*-trend |
|---|---|---|---|---|---|---|---|
| α-Carotene | | | | | | | |
| Median intake (mg/day) | | 0.19 | 0.38 | 0.57 | 0.82 | 1.31 | |
| RR (95% CI)† | 569 | 1.00 | 0.85 (0.63–1.13) | 0.74 (0.55–1.00) | 0.90 (0.68–1.20) | 0.83 (0.61–1.11) | 0.27 |
| RR (95% CI)‡ | 475 | 1.00 | 0.81 (0.58–1.13) | 0.76 (0.54–1.06) | 0.83 (0.60–1.16) | 0.99 (0.71–1.39) | 0.89 |
| β-Carotene | | | | | | | |
| Median intake (mg/day) | | 1.43 | 2.10 | 2.65 | 3.37 | 4.76 | |
| RR (95% CI)† | 569 | 1.00 | 1.04 (0.77–1.39) | 0.87 (0.64–1.18) | 0.89 (0.66–1.20) | 0.98 (0.73–1.32) | 0.51 |
| RR (95% CI)‡ | 475 | 1.00 | 1.07 (0.77–1.49) | 0.90 (0.63–1.27) | 0.93 (0.66–1.32) | 1.16 (0.82–1.63) | 0.67 |
| Lutein and zeaxanthin | | | | | | | |
| Median intake (mg/day) | | 1.34 | 1.86 | 2.34 | 2.84 | 3.82 | |
| RR (95% CI)† | 569 | 1.00 | 0.97 (0.72–1.31) | 0.86 (0.64–1.16) | 0.84 (0.62–1.13) | 1.02 (0.76–1.37) | 0.73 |
| RR (95% CI)‡ | 475 | 1.00 | 1.00 (0.71–1.41) | 0.89 (0.63–1.26) | 0.99 (0.70–1.40) | 1.11 (0.79–1.57) | 0.49 |
| β-cryptoxanthin | | | | | | | |
| Median intake (mg/day) | | 0.02 | 0.06 | 0.13 | 0.25 | 0.40 | |
| RR (95% CI)† | 569 | 1.00 | 0.63 (0.48–0.83) | 0.54 (0.40–0.72) | 0.59 (0.44–0.80) | 0.71 (0.53–0.95) | < 0.01 |
| RR (95% CI)‡ | 475 | 1.00 | 0.70 (0.52–0.95) | 0.56 (0.40–0.78) | 0.62 (0.44–0.87) | 0.74 (0.53–1.03) | < 0.01 |
| Lycopene | | | | | | | |
| Median intake (mg/day) | | 0.15 | 0.48 | 0.81 | 1.21 | 2.21 | |
| RR (95% CI)† | 569 | 1.00 | 0.83 (0.62–1.11) | 1.07 (0.81–1.42) | 1.05 (0.78–1.41) | 0.97 (0.72–1.31) | 0.58 |
| RR (95% CI)‡ | 475 | 1.00 | 0.85 (0.61–1.18) | 1.07 (0.77–1.48) | 1.18 (0.84–1.65) | 1.08 (0.77–1.51) | 0.16 |

*Reference category; †adjusted for age (years) and sex; ‡adjusted for age (years), sex, cigarette smoking amount (cig/day) and cigarette smoking duration (years).

for these covariates increased the risk estimates not essentially (Table 2). Also, adjustment for other potential confounders did not substantially change the risk estimates (data not shown).

The intake of the carotenoids α-carotene, β-carotene, luteine/zeaxantine, and lycopene were not associated with bladder cancer risk (Table 3). The intake of β-cryptoxanthin appeared to be inversely associated with bladder cancer risk ($p$-trend < 0.01) with corresponding age- and sex adjusted RRs per increasing quintiles of intake of 1.00 (reference), 0.63 (CI: 0.48–0.83), 0.54 (CI: 0.40–0.72), 0.59 (CI: 0.44–0.80) and 0.71 (CI: 0.53–0.95), respectively. Adjustment for cigarette smoking or other potential confounders did not change the results substantially (Table 3).

Since the intake of vitamin C and β-cryptoxanthin were correlated ($r$ = 0.69), and both vitamin C and β-cryptoxanthin were inversely associated with bladder cancer risk (Tables 2 and 3) we estimated the incidence rate ratios for both nutrients after adjustment for each other in addition to age, sex, cigarette smoking amount and cigarette smoking duration (Table 4). After adjustment for β-cryptoxanthin intake, the inverse association for vitamin C intake disappeared. However, the RRs for β-cryptoxanthin intake did not change essentially after adjustment for vitamin C intake (Table 4).

We investigated potential effect modification of the association between β-cryptoxanthin and bladder cancer risk by cigarette smoking amount and duration (Table 5). We found a statistically significant interaction effect with smoking amount ($P$ < 0.01) but not with smoking duration ($P$ = 0.10). The inverse association between β-cryptoxanthin and bladder cancer risk was not found among non-smokers and appeared to be limited to cigarette smokers who smoked more than 15 cigarettes per day or who smoked for more than 35 years (Table 5)

The protective effect of β-cryptoxanthin intake was observed in each stratum of tumour invasiveness or morphology of male transitional cell carcinomas of the bladder, although the association was less pronounced for non-invasive papillary tumours (Table 6).

**Table 4** Incidence rate ratios and 95% confidence intervals for bladder cancer according to quintiles of vitamins C and β-cryptoxanthin adjusted for each other, Netherlands Cohort Study (1986–1992)

| | Quintiles of intake 1 (low)* | 2 | 3 | 4 | 5 (high) | *P*-trend |
|---|---|---|---|---|---|---|
| Vitamin C† | | | | | | |
| RR (95% CI)‡ | 1.00 | 1.08 (0.80–1.46) | | 1.07 (0.73–1.57) | | 0.67 |
| β-cryptoxanthin | | | | | | |
| RR (95% CI)§ | 1.00 | 0.69 (0.50–0.95) | 0.54 (0.38–0.78) | 0.60 (0.40–0.89) | 0.71 (0.46–1.11) | 0.01 |

*Reference category; †for vitamin C, quintiles 2 and 3 and quintiles 4 and 5 were combined; ‡adjusted for age (years), sex, cigarette smoking amount (cig/day), cigarette smoking duration (years) and β-cryptoxanthin intake; §adjusted for age (years), sex, cigarette smoking amount (cig/day), cigarette smoking duration (years) and vitamin C intake.

 *© 2001 Cancer Research Campaign*

**Table 5** Incidence rate ratios and 95% confidence intervals for bladder cancer according to β-cryptoxanthin intake, with respect to cigarette smoking amount and duration; Netherlands Cohort Study (1986–1992)

| | Quintiles of β-cryptoxanthin intake | | | | | |
|---|---|---|---|---|---|---|
| **Cigarette smoking** | **1 (low)*** | **2** | **3** | **4** | **5 (high)** | ***P*-trend** |
| Never-smoker† | 1.00 | 0.86 (0.35–2.13) | 0.88 (0.37–2.09) | 0.89 (0.38–2.08) | 1.16 (0.51–2.64) | 0.67 |
| Amount (cig/day)‡ | | | | | | |
| < 15 | 1.00 | 0.83 (0.50–1.40) | 0.59 (0.33–1.06) | 0.98 (0.57–1.67) | 1.34 (0.79–2.27) | 0.25 |
| 15+ | 1.00 | 0.68 (0.47–1.00) | 0.59 (0.39–0.88) | 0.50 (0.32–0.78) | 0.54 (0.35–0.83) | < 0.01 |
| Duration (years)§ | | | | | | |
| < 35 | 1.00 | 0.83 (0.49–1.39) | 0.52 (0.29–0.94) | 0.71 (0.40–1.26) | 0.90 (0.52–1.54) | 0.47 |
| 35+ | 1.00 | 0.67 (0.46–0.99) | 0.61 (0.41–0.91) | 0.60 (0.39–0.91) | 0.67 (0.43–1.03) | < 0.01 |

*Reference category; †adjusted for age and sex; ‡adjusted for age (years), sex, and cigarette smoking duration (years); §adjusted for age (years), sex, and cigarette smoking amount (cig/day).

The risks of bladder cancer for consumers of vitamin supplements containing vitamin A, vitamin C or Vitamin E were similar to the risk for non-consumers. After adjustment for age, sex, cigarette smoking amount and cigarette smoking duration the RRs were 1.12 (CI: 0.71–1.78), 1.01 (CI: 0.69–1.48), and 1.05 (CI: 0.63–1.73), respectively (data not shown).

### DISCUSSION

The results of the prospective Netherlands Cohort Study showed that, in general, the intake of retinol, vitamin C, vitamin E, folate, carotenoids, and the use of vitamin supplements are not associated with bladder cancer risk. The initially inverse association with vitamin C intake disappeared after controlling for β-cryptoxanthin intake. Only β-cryptoxanthin intake appeared to be inversely associated with bladder cancer risk, especially among heavy cigarette smokers.

The NLCS was carried out in the general Dutch population of men and women aged 55–69 years at baseline. After 6.3 years of follow-up, 569 bladder cancer cases were detected which is substantially more than any other prospective study that investigated the association between these nutrients, and the risk of bladder cancer. The prospective nature of a cohort study together with completeness of follow-up, as has been achieved in this study, reduced the potential for selection bias to a minimum. Non-differential misclassification of exposure, may have resulted in underestimation of the strength of the association. However, the results of the validation study showed that the questionnaire was able to rank subjects adequately according to their intake of most nutrients. In a reproducibility study, it was further demonstrated that the single food frequency questionnaire measurement could characterize dietary habits for a period of at least 5 years (Goldbohm et al, 1995). Information bias is also largely avoided because dietary habits were reported before bladder cancer was diagnosed. A change in dietary habits of subjects with latent bladder cancer at the time of completing the baseline questionnaire is much less likely than in subjects with, e.g. gastrointestinal cancer. Furthermore, repeating analyses after excluding cases diagnosed in the first 1 or 2 years hardly affected our results.

A potentially more realistic problem in evaluating the observed inverse associations is residual confounding by cigarette smoking. We modelled cigarette smoking habits such that they best explained bladder cancer (Zeegers et al, 2000a). This resulted in a model including number of years smoked and habitual number of cigarettes smoked per day, both as continuous variables. We therefore believe that the associations observed were not entirely due to residual confounding by smoking, although we cannot exclude some influence. When we added the smoking variables to an age- and sex-adjusted model, the RR estimates changed only slightly. We were not able to explain our results on the basis of confounding of other factors in addition to cigarette smoking, since our results were essentially unchanged after incorporating into the analyses many known or suspected risk factors for bladder cancer, including total consumption of alcohol, coffee, tea, and water, high risk occupation, and family history of bladder cancer.

**Table 6** Incidence rate ratios and 95% confidence intervals for transitional cell carcinoma of the urinary bladder according to β-cryptoxanthin intake, with respect to tumour invasiveness and morphology; Netherlands Cohort Study (1986–1992)

| | Non-invasive ($T_{IS}/T_s/T_1$) | | Invasive ($T_{2–4}$) | |
|---|---|---|---|---|
| **β-cryptoxanthin (quintiles of intake)** | **Non-papillary (n = 24)* RR (95% CI)‡** | **Papillary (n = 232)† RR (95% CI)‡** | **Non-papillary (n = 111)* RR (95% CI)‡** | **Papillary (n = 146)† RR (95% CI)‡** |
| 1 (low) | 1.00 (reference) | 1.00 (reference) | 1.00 (reference) | 1.00 (reference) |
| 2 | 0.38 (0.10–1.43) | 1.03 (0.68–1.57) | 0.66 (0.37–1.19) | 0.51 (0.29–0.88) |
| 3 | 0.30 (0.06–1.42) | 0.75 (0.47–1.20) | 0.54 (0.28–1.02) | 0.44 (0.24–0.79) |
| 4 | 0.51 (0.14–1.88) | 0.82 (0.51–1.33) | 0.55 (0.28–1.09) | 0.49 (0.27–0.88) |
| 5 (high) | 0.18 (0.02–1.57) | 0.84 (0.52–1.36) | 0.72 (0.38–1.36) | 0.71 (0.41–1.23) |
| *P*-value for linear trend | 0.04 | 0.22 | 0.11 | 0.06 |

ICD-O:M8120, †ICDO:M8130, ‡adjusted for age (years), sex, cigarette smoking amount (cig/day), and cigarette smoking duration (years).

© 2001 Cancer Research Campaign 

Vitamin A is important for cell differentiation and since lack of differentiation is a feature of cancer cells, a protective effect of vitamin A on cancer development may be expected (Kamat and Lamm, 1999; Patterson et al, 1997; Steinmetz and Potter, 1991). Retinol is the physiologically active form of vitamin A and can also be derived from some carotenoids (e.g. α-carotene, β-carotene, and β-cryptoxantine). Some carotenoids have antioxidant properties and may inhibit carcinogenesis by preventing DNA damage induced by free radicals. However, a recently published meta-analysis based on seven case control studies and three cohort studies did not find increased risks for diets low in retinol or β-carotene (Steinmaus et al, 2000). A prospective study that was published more recently also found no association between supplemental and dietary vitamin A intake and bladder cancer risk (Michaud et al, 2000). In the present study, only the dietary intake of the carotenoid β-cryptoxanthin appeared to be inversely associated with bladder cancer risk. We know of no biological mechanism other than its provitamin A activity that might explain this specific association. Citrus fruits are the main sources of β-cryptoxanthin. In a previous analysis we also presented an inverse association between citrus fruit consumption and bladder cancer risk (Zeegers, submitted for publication). However, an other previously conducted prospective study did not find an association between β-cryptoxanthin and bladder cancer risk (Michaud et al, 1999). Further research is needed to evaluate this matter.

Vitamin C is mostly known for its antioxidant capacity (Steinmetz and Potter, 1991) and its ability to prevent formation of nitrosamine and other N-nitroso compounds (Birt, 1986). In addition to β-cryptoxanthin, citrus fruits are also known for their high content of vitamin C. After adjustment for β-cryptoxanthin, the small inverse association between vitamin C intake and bladder cancer risk increased towards unity. Two previously conducted population based case-control studies found small increased risks of bladder cancer with increasing intake of dietary vitamin C for men and women combined (Risch et al, 1988) or for men only (Nomura et al, 1991) after controlling for cigarette smoking. However, other population based case control studies presented decreased risks for men and women combined (Bruemmer et al, 1996) or for women only (Nomura et al, 1991), or found no association at all (Riboli et al, 1991). One prospective study suggested an inverse association with dose of vitamin C supplement use, but not with dietary vitamin C intake (Michaud et al, 2000), which is in correspondence to the results of the present study.

Like vitamin C, vitamin E is an intracellular antioxidant and can inhibit nitrosation (Kamat and Lamm, 1999; Knekt et al, 1988; Patterson et al, 1997). Two population based case control studies (Bruemmer et al, 1996; Riboli et al, 1991) and one prospective study (Michaud et al, 2000) reported inverse associations between dietary vitamin E intake and bladder cancer risk. We did not find statistically significant associations, although the point estimates were moderately negative.

Folate is essential for methylation reactions in the human body. Reduced methylation of DNA may contribute to loss of normal controls on proto-oncogene expression. Folate deficiency causes massive incorporation of uracil into human DNA and chromosome breaks. Such breaks could contribute to an increased risk of cancer (Blount et al, 1997; Glynn and Albanes, 1994). The epidemiologic evidence relating dietary folate and the risk of bladder cancer is limited. One previously conducted case-control study found a moderately inverse association between folate intake and bladder cancer risk (Bruemmer et al, 1996), whereas a recently published prospective study did not find an association with bladder cancer risk (Michaud et al, 2000). In the present study, we found no association between folate intake and bladder cancer risk.

We conclude that dietary or supplemental intake of vitamin A, vitamin C, vitamin E, and dietary intake from folate and carotenoids are not associated with bladder cancer risk. Only β-cryptoxanthin appeared to be inversely associated and this deserves further scientific attention.

### ACKNOWLEDGEMENTS

The Netherlands Cohort Study was supported by the Dutch Cancer Society. The authors thank the regional cancer registries, the Dutch national database of pathology reports (PALGA), and the National Health Care Information Center for providing incidence data; S. van de Crommert, H. Brants, W. van Dijk, M. Moll, J. Nelissen, C. de Zwart, A. Pisters, H. van Montfort, R. Schmeitz, T. van Montfort, and T. van Moergastel for assistance.

### REFERENCES

Barlow WE (1994) Robust variance estimation for the case-cohort design. *Biometrics* **50**: 1064–1072

Birt DF (1986) Update on the effects of vitamins A, C, and E and selenium on carcinogenesis. *Proc Soc Exp Biol Med* **183**: 311–320

Blount BC, Mack MM, Wehr CM, MacGregor JT, Hiatt RA, Wang G, Wickramasinghe SN, Everson RB and Ames BN (1997) Folate deficiency causes uracil misincorporation into human DNA and chromosome breakage: implications for cancer and neuronal damage. *Proc Natl Acad Sci USA* **94**: 3290–3295

Bruemmer B, White E, Vaughan TL and Cheney CL (1996) Nutrient intake in relation to bladder cancer among middle-aged men and women. *Am J Epidemiol* **144**: 485–495

Chyou PH, Nomura AM and Stemmermann GN (1993) A prospective study of diet, smoking, and lower urinary tract cancer. *Ann Epidemiol* **3**: 211–216

D'Avanzo B, La Vecchia C, Negri E, Decarli A and Benichou J (1995) Attributable risks for bladder cancer in northern Italy. *Ann Epidemiol* **5**: 427–431

Glynn SA and Albanes D (1994) Folate and cancer: a review of the literature. *Nutr Cancer* **22**: 101–119

Goldbohm RA, Brants HA, Hulshof KF and van den Brandt PA (1998) The contribution of various foods to intake of vitamin A and carotenoids in The Netherlands. *Int J Vitam Nutr Res* **68**: 378–383

Goldbohm RA, van den Brandt PA, Brants HA, van het Veer P, Al M, Sturmans F and Hermus RJSO (1994a) Validation of a dietary questionnaire used in large-scale prospective cohort study on diet and cancer. *Eur J Clin Nutr* **48**: 253–265

Goldbohm RA, van den Brandt PA and Dorant E (1994b) Estimation of the coverage of Dutch municipalities by cancer registries and PALGA based on hospital discharge data. *Tijdschr Soc Gezond* **72**: 80–84

Goldbohm RA, van 't Veer P, van den Brandt PA, van 't Hof MA, Brants HA, Sturmans F and Hermus RJ (1995) Reproducibility of a food frequency questionnaire and stability of dietary habits determined from five annually repeated measurements. *Eur J Clin Nutr* **49**: 420–429

Johansson SL and Cohen SM (1997) Epidemiology and etiology of bladder cancer. *Semin Surg Oncol* **13**: 291–298

Kamat AM and Lamm DL (1999) Chemoprevention of urological cancer. *J Urol* **161**: 1748–1760

Knekt P, Aromaa A, Maatela J, Aaran RK, Nikkari T, Hakama M, Hakulinen T, Peto R, Saxen E and Teppo L (1988) Serum vitamin E and risk of cancer among Finnish men during a 10-year follow-up. *Am J Epidemiol* **127**: 28–41

Konings EJ (1999) A validated liquid chromatographic method for determining folates in vegetables, milk powder, liver, and flour. *J AOAC Int* **82**: 119–127

Lin DY and Ying Z (1993) Cox regression with incomplete covariate measurements. *JASA* **88**: 1341–1349

Michaud DS, Spiegelman D, Clinton SK, Rimm EB, Willett WC and Giovannucci E (2000) Prospective study of dietary supplements, macronutrients, micronutrients, and risk of bladder cancer in US men. [In Process Citation]. *Am J Epidemiol* **152**: 1145–1153

 *© 2001 Cancer Research Campaign*

Michaud DS, Spiegelman D, Clinton SK, Rimm EB, Willett WC and Giovannucci EL (1999) Fruit and vegetable intake and incidence of bladder cancer in a male prospective cohort. *J Nutr Cancer Inst* **91**: 605–613

Mills, PK, Beeson WL, Phillips RL and Fraser GE (1991) Bladder cancer in a low risk population: results from the Adventist Health Study. *Am J Epidemiol* **133**: 230–239

Negri E, La Vecchia C, Franceschi SBDA and Parazzini F (1991) Vegetable and fruit consumption and cancer risk. *Int J Cancer* **48**: 350–354

NEVO-table (1986) *Dutch food composition table 1986–1987*. Voolichtingsbureau voor de Voeding: The Hague, Netherlands

Nomura AMY, Kolonel LN, Hankin JH and Yoshizawa CN (1991) Dietary factors in cancer of the urinary tract. *Int J Cancer* **48**: 199–205

Parkin DM, Pisani P and Ferlay J (1999) Estimates of the worldwide incidence of 25 major cancers in 1990. *Int J Cancer* **80**: 827–841

Patterson RE, White E, Kristal AR, Neuhouser ML and Potter JD (1997) Vitamin supplements and cancer risk: the epidemiologic evidence. *Cancer Causes Control* **8**: 786–802

Prentice RL (1986) A case-cohort design for epidemiologic cohort studies and disease prevention trials. *Biometrika* **73**: 1–11

Riboli E, Gonzalez CA, Lopez Abente G, Errezola M, Izarzugaza I, Escolar A, Nebot M, Hemon B and Agudo A (1991) Diet and bladder cancer in Spain: a multi-centre case-control study. *Int J Cancer* **49**: 214–219

Risch HA, Burch JD, Miller AB, Hill GB, Steele R and Howe GR (1988) Dietary factors and the incidence of cancer of the urinary bladder. *Am J Epidemiol* **127**: 1179–1191

Ross RK, Jones PA and Yu MC (1996) Bladder cancer epidemiology and pathogenesis. *Semin Oncol* **23**: 536–545

Shibata A, Paganini Hill A, Ross RK and Henderson BE (1992) Intake of vegetables, fruits, beta-carotene, vitamin C and vitamin supplements and cancer incidence among the elderly: a prospective study. *Br J Cancer* **66**: 673–679

Silverman DT, Hartge P, Morrison AS and Devesa SS (1992) Epidemiology of bladder cancer. *Hematol Oncol Clin North Am* **6**: 1–30

StataCorp (1999) *Stata Statistical Software: Release 6.0*. Stata Corporation: College Station, TX

Steinmaus CM, Nunez S and Smith AH (2000) Diet and bladder cancer: a meta-analysis of six dietary variables. *Am J Epidemiol* **151**: 693–702

Steinmetz KA and Potter JD (1991) Vegetables, fruit, and cancer. II. Mechanisms. *Cancer Causes Control* **2**: 427–442

van den Brandt PA, Goldbohm RA, van het Veer PAV, Hermus RJJ and Sturmans F (1990a) A large-scale prospective cohort study on diet and cancer in the Netherlands. *J Clin Epidemiol* **43**: 285–295

van den Brandt PA, Schouten LJ, Goldbohm RA, Dorant E and Hunen PHM (1990b) Development of a record linkage protocol for use in the Dutch cancer registry for epidemiological research. *Int J Epidemiol* **19**: 553–558

van der Meijden APM (1998) Bladder cancer. *BMJ* **317**: 1366–1369

Volovics A and van den Brandt PA (1997) Methods for the analyses of case-cohort studies. *Biometrical Journal* **2**: 195–214

World Cancer Research Fund & American Institute for Cancer Research (1997) Bladder. In: *Food, Nutrition and the Prevention of Cancer: a global perspective* pp. 338–61. Banta Book Group: Menasha

Zeegers MP, Tan FE, Verhagen AP, Weijenberg MP and van den Brandt PA (1999) Elevated risk of cancer of the urinary tract for alcohol drinkers: a meta-analysis. *Cancer Causes Control* **10**: 445–451

Zeegers MP, Tan FE, Dorant E and van den Brandt PA (2000a) The impact of characteristics of cigarette smoking on urinary tract cancer risk: a meta-analysis of epidemiologic studies. *Cancer* **89**: 630–639

Zeegers MPA, Tan FES, Goldbohm RA and van den Brandt PA (2000b) Are coffee and tea consumption associated with urinary tract cancer risk: a systematic review and meta-analysis. *Int J Epidemiol* in press

*© 2001 Cancer Research Campaign*
